# Pre-Crystallization of Nougat by Seeding with Cocoa Butter Crystals Enhances the Bloom Stability of Nougat Pralines

**DOI:** 10.3390/foods10051056

**Published:** 2021-05-11

**Authors:** Birgit Böhme, Annika Bickhardt, Harald Rohm

**Affiliations:** Chair of Food Engineering, Institute of Natural Materials Technology, Technische Universität Dresden, 01062 Dresden, Germany; birgit.boehme@tu-dresden.de (B.B.); annika_sontje.bickhardt@tu-dresden.de (A.B.)

**Keywords:** praline, nougat, chocolate, fat bloom, DSC, hardness, crystal seeding, migration

## Abstract

Fat bloom is an outstanding quality defect especially in filled chocolate, which usually comprises oils of different origins and with different physical properties. Dark chocolate pralines filled with nougat contain a significant amount of hazelnut oil in their center and have been reported as being notably susceptible to oil migration. The current study was designed to test the assumption that a targeted crystallization of nougat with cocoa butter seed crystals is an appropriate technological tool to reduce filling oil transfer to the outside of the praline and, hence, to counteract chocolate shell weakening and the development of fat bloom. For this purpose, the hardness of nougat/chocolate layer models and the thermal properties of chocolate on top of nougat were analyzed during storage at 23 °C for up to 84 days. Pronounced differences between layer models with seeded nougat and with control nougat that was traditionally tempered were observed. The facts that chocolate hardness increased rather than decreased during storage, that the cocoa butter melting peak was shifted towards a lower temperature, and that the hazelnut oil content in the chocolate was reduced can be taken as explicit indicators for the contribution of seeded nougat to the fat bloom stability of filled chocolate.

## 1. Introduction

One of the key quality attributes of dark chocolate is its appearance and, particularly, a smooth and glossy surface. Fat bloom is a prominent appearance defect that results in a loss of gloss and that devaluates the products by turning their surface dull and greyish. The main driving force behind fat bloom is the transformation of ßV cocoa butter crystals into the ßVI polymorph which, for instance, occurs when a small amount of cocoa butter is released from the chocolate matrix and recrystallizes at the surface [1,2,3]. In plain chocolate, the fat bloom is mainly triggered by (a) insufficient environmental conditions, for instance, pronounced temperature fluctuations or a storage temperature higher than the melting point of the ßV polymorph, or by (b) poor tempering during production so that the necessary crystal nuclei cannot be generated [4,5]. The situation becomes even more complicated when fats or oils other than cocoa butter are present in the system. These may either be comprised in the chocolate formulation or may be part of a second system that is in close contact with the chocolate.

The latter is true in the case of filled chocolate confectionery, for instance, pralines. A prominent and frequently consumed example is pralines filled with dark "Viennese" nougat. Similar to chocolate, nougat represents an oil-based multiphase suspension, made of different constituents. The main ingredients are hazelnuts and sugar which maybe, depending on the formulation, supplemented by a certain amount of cocoa butter and/or cocoa mass, milk solids including milk fat, and surface-active compounds. The fat content of nougat is typically in a range of 30–45 g/100 g and, to a large extent, made of hazelnut oil [6]. Nougat processing comprises the milling of roasted hazelnuts together with sugar and the other ingredients, traditionally in edge mills and, nowadays, mainly in five roller refiners or in agitated ball mills. Hazelnut oil differs largely from cocoa butter with respect to fatty acid and, hence, its triacylglycerol composition. As a consequence, the melting point and the solid fat content at a specific temperature also differ, and significant interactions between the fat phases of nougat and the surrounding chocolate can be expected.

The driving force behind fat bloom in confectionery products with different fats is migration [7]. The concentration difference at the interface between the chocolate shell and the filling induces a diffusive transport of the filling oil through the chocolate shell towards its outside, especially when the filling is rich in unsaturated fatty acids. Due to the higher mobility of the unsaturated oil, metastable cocoa butter crystals dissolve in the migrating oil and recrystallize at the surface as stable ßVI [8]. Apart from geometrical considerations, it has been reported that milk fat or some vegetable fats included in chocolate may help to decelerate fat bloom [9], or that specific barrier layers between chocolate and filling can be successfully introduced as a third component [10,11].

Seeding with cocoa butter crystals is an established alternative to the traditional stir/shear temper technology of chocolate [12]. Especially when containing cocoa butter, nougat also needs to be tempered during processing, regularly by applying the stir/shear technology. The current study aimed to evaluate whether a targeted pre-crystallization of dark nougat with cocoa butter crystals could be an innovative technology to enhance the long-term physical stability of filled confectionery products.

## 2. Materials and Methods

### 2.1. Materials

Dark chocolate mass, made of sugar, cocoa mass, cocoa butter, and soy lecithin, with a fat content of 32.4 g/100 g, was obtained from a German chocolate manufacturer. Four commercial nougat samples, encoded #A, #B, #C, and #D, and the respective formulations (Table 1) were obtained from two companies. SEED 100 cocoa butter crystals (ßV) were provided by Uelzena eG (Uelzen, Germany). The melting peak temperature measured by DSC was 34.0 ± 0.1 °C and the phase transition enthalpy was 132.5 ± 0.7 J/g, which is in line with literature data on the ßV polymorph [13,14] (Figure S1). It needs to be mentioned that other researchers (e.g., [12,15] associated such a melting temperature with polymorph ßVI, but this will not be discussed further. The seed crystal powder has a mean particle size of approx. 25 µm [16], which was confirmed by light microscopy (Figure S2). All chemicals used in the study were of analytical grade.

### 2.2. Production and Storage of Nougat/Chocolate Layer Models

Layer models were produced to ensure controlled contact between nougat and chocolate. For this purpose, 16.0 ± 0.2 g liquid nougat was transferred into Petri dishes of 55 mm diameter, manually compacted by vibration, and solidified at 18 °C for 7 d in an environmental chamber. This nougat layer had a thickness of approx. 5 mm. The following samples were prepared: (a) Control plates were made at the nougat manufacturer sites after stir/shear tempering and, after solidification as specified above, sent to the laboratory. (b) For the experimental plates, nougat was melted at 50 °C overnight, mixed, and then cooled to 27 °C under continuous stirring. The amount of ßV cocoa butter crystals subsequently added was either 0.5%, 1.0%, or 2.0%, as related to the total fat content of the nougats. The average temper degree of the nougat immediately before weighing into the plates was determined using an E3 tempermeter (Sollich KG, Bad Salzuflen, Germany). The temper degree was 5.1 ± 0.2 when seeded with 0.5% crystals, 5.8 ± 0.3 when seeded with 1%, and 6.6 ± 0.3 when seeded with 2% cocoa butter crystals.

After solidification, the nougat plates were overlaid with 6.0 ± 0.2 g tempered chocolate, resulting in a layer of approx. 2 mm thickness. The chocolate used for this purpose was melted and conditioned to 50 °C overnight and tempered at 31 °C using a Minitemper^®^ Turbo (Sollich KG, Bad Salzuflen, Germany). The final layer models were solidified at 18 °C for 24 h and subsequently stored at 23 ± 0.2 °C for up to 84 d in an environmental chamber. Individual samples of the layer models were analyzed after 1, 7, 21, 28, 63, and 84 d.

### 2.3. Methods for the Analysis of Raw Materials

Particle size distributions were analyzed using a HELOS KR laser diffractometer (Sympatec GmbH, Clausthal-Zellerfeld, DE). Prior to analysis, a 2 g sample conditioned to 50 °C was suspended in 10 g sunflower oil of the same temperature in duplicate [17]. Measurements were carried out in a 50 mL cuvette filled with sunflower oil to which the sample suspension was dropwise added until an optical density of approx. 30% was achieved. From the volume-specific density distribution the x_10_, x_50_, and x_90_ diameters, representing the 10%, 50%, and 90% quantiles, respectively, were taken.

The amount of mobile fat was determined using a centrifugation method [18]. Fourteen g melted nougat was transferred into conical tubes and centrifuged at 35 °C for 15 min at 10,200 g (Heraeus Biofuge Stratos, Kendro Laboratory Products GmbH, Langenselbold, Germany). The amount of separated fat was weighed and represents the mobile fat, expressed as a gravimetric fraction of total fat.

Viscosity measurements were carried out with a MARS III rheometer equipped with a concentric CC25DIN geometry (r_i_ = 12.54 mm, r_o_ = 13.60 mm, h = 37.62 mm; Thermo Fisher GmbH, Karlsruhe, Germany). The temperature was kept constant at 40 °C using a Peltier device. After equilibration and pre-shearing for 300 s at 5/s, the shear rate was increased from 2/s to 50/s within 180 s, kept constant at 50/s for 60 s, and then reduced to 2/s in another 180 s [19]. Fifty data points per ramp were recorded in logarithmic spacing. The downward cycle was used for fitting the shear stress—shear rate data to the Casson model, and Casson yield stress and viscosity were taken as descriptors.

### 2.4. Methods for the Analysis of Raw Materials and Layer Models

The hardness of base chocolate, nougat, and chocolate/nougat layers was measured using a TA.XTplus texture analyzer (Stable Micro Systems Ltd., Surrey, UK) equipped with a 50 N force transducer [20]. By applying a crosshead velocity of 1 mm/s, samples were penetrated with a 2 mm diameter plunger. The maximum penetration depth was 2 mm. The maximum force was taken as a hardness indicator and calculated as an arithmetic mean of 8 replicate measurements (2 Petri dishes × 4 measurements per petri dish).

Thermal properties of chocolate were determined in duplicate with a DSC25 instrument connected to an RCS90 cooling unit (TA Instruments GmbH, Eschborn, Germany). Using an analytical balance, 5 ± 0.2 mg sample scratched from the surface of the layer models was transferred into a standard aluminum pan and closed. An empty pan served as a reference. After cooling to −50 °C and stabilization for 3 min, the samples were heated to 45 °C at 10 K/min under a continuous nitrogen flow of 50 mL/min. The thermograms were evaluated with respect to cocoa butter melting peak temperature and the enthalpy of the nut oil melting peak, which was calculated from the heat flow function.

### 2.5. Sensory Analysis

A duo-trio forced-choice test setup was used to evaluate whether stir/shear tempered and seeded nougat can be distinguished by tasting. For this purpose, 6 g aliquots of tempered or seeded nougat were dosed into paper cupcakes with an upper diameter of 35 mm and a height of 20 mm. The samples were then stored for 7–10 d at 18 °C.

A panel of 10 trained panelists took part in the study. Informed consent was obtained from all subjects involved in the study. Due to CoVid-19 restrictions, author A.B. who was in charge of the experiments delivered triads of the samples to the individual offices of the subjects. Samples were presented in Petri dishes encoded with 3-digit random numbers, and the panelists were asked to identify the odd specimen. The number of correct identifications was judged for significance using the tables of Roessler et al. [21].

### 2.6. Statistics

Statistical data evaluation was carried out using SAS^®^ Studio 3.8 University Edition (SAS Institute Inc., Cary, NC, USA). Outliers were identified with the Dixon-Q test at *p* < 0.05. One-way analysis of variance was followed by Fisher’s least significant difference post-hoc tests. All significance statements given in this study refer to an error probability level of *p* < 0.05.

## 3. Results and Discussion

### 3.1. Description of the Base Nougat

The total fat content of the base nougats ranged from 36.0–44.0 g/100 g (Table 2). The relative amount of cocoa butter in the oil fraction of the nougats was calculated based on an approximate fat content of the cocoa mass (53 g/100 g) and the amount of cocoa butter in the formulation, and ranged between 25.2% and 29.5% (nougat #A, #B, #C) but was 41.7% in case of nougat #D. The fraction of mobile fat was significantly higher for samples #A and #B than for nougat #D (approx. 26% vs. 18.5%, respectively). As for chocolate, the flow properties of liquid nougat depend on the volume fraction and surface properties of dispersed particles and are additionally affected by the presence of surface-active compounds [22,23]. Casson yield stress and viscosity were lowest for nougat #B which had the highest fat content and, hence, the lowest particle load, and highest for #D which showed the lowest fat content and, additionally, the lowest amount of mobile fat. Nougat hardness measured by penetration was approx. 1 N (samples #A, #B, #C) but significantly higher for nougat #D (3.76 ± 0.32 N).

**Figure 1:**
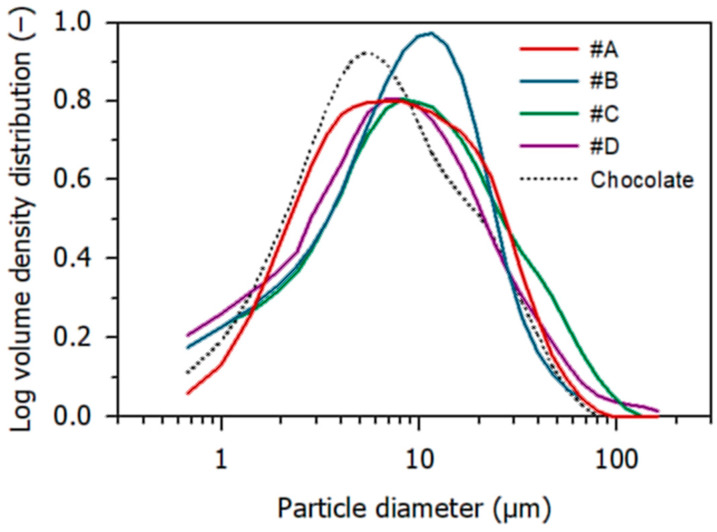
Particle size distributions of nougat samples #A–#D and of the chocolate used for the preparation of the layer models.

Figure 1 displays the logarithmic volume-based particle size distributions, which were monomodal for all samples. The particle diameter median (x_50,3_) was 6.1 µm for the chocolate, and slightly higher (7.4–8.5 µm) for the four nougats. The span between the x_10,3_ and the x_90,3_ quantiles was 22.4 µm and 21.8–33.6 µm for chocolate and nougat, respectively. The highest x_90,3_ diameter (35.1 µm) was observed in the case of nougat #C, which is slightly above the critical particle size for chocolate concerning the sensory perception of graininess [24,25]. This value was, however, recently questioned by de Pelsmaeker et al. [26].

It is evident from the data that the nougat samples are representative of what is available on the market, starting with the high fat, low viscosity variant #B comprising of only hazelnuts, sugar, and cocoa butter. The fraction of dispersed particles for all other nougats were explicitly higher, with some also containing milk solids and/or cocoa mass and/or surface-active compounds such as lecithin at varying concentration.

### 3.2. Storage Induced Changes and Nut Oil Migration

**Figure 2:**
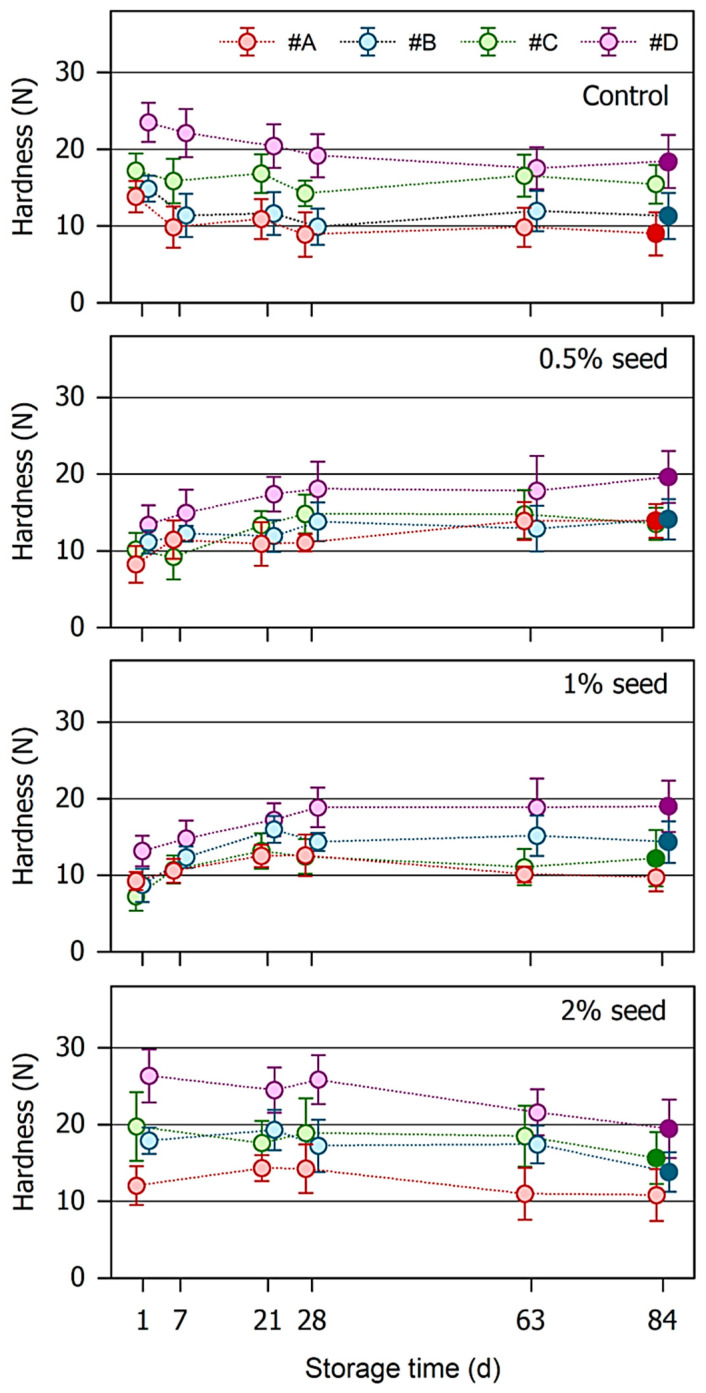
Development of the hardness of the nougat/chocolate layer models during storage at 23 °C for 84 days. Four different nougats (#A, #B, #C, #D) were either tempered using the stir/shear technique (control) or seeded with different amounts of cocoa butter crystals. For the sake of clarity, symbols are slightly shifted along the x-axis. Data are arithmetic mean ± standard deviation of 8-fold measurements. The hardness of samples at 84 days marked by a filled symbol is significantly (*p* < 0.05) different from the hardness of the fresh layer models.

Figure 2 depicts the development of the hardness of the surface chocolate during storage as affected by nougat variety and pre-crystallization method and intensity. One day after production of the layer model, the hardness of the chocolate on top of the nougat was 13.9 ± 1.8 N for sample #A, 14.9 ± 1.8 N for #B, and 17.2 ± 2.1 N for #C. The mechanical response of nougat #D was 23.5 ± 2.3 N, thus reflecting the contribution of the stiffer bottom layer (see nougat hardness in Table 2) to chocolate hardness.

During storage at 23 °C, the hardness of the chocolate of the control sample decreased, especially within the first four weeks. After that, further changes in sample hardness were only minor. It is also evident that the relative deviation of the repeated measurements of individual samples increased with storage time. This can be attributed to local effects and increasing differences between local spots at the surface of the layer models. As indicated in the chart, the softening induced by 84 d storage was significant for chocolate on top of nougat #A, #B, and #D.

The situation is, however, different when cocoa butter crystals were used to induce pre-crystallization in nougat. In case an amount of 0.5% or 1% seed powder was used for seeding, initial chocolate hardness was approx. 10 N, with the highest values again for samples with nougat #D. In the first 20–30 d of storage, the systems slightly solidified, presumably because of an ongoing slow crystallization of cocoa butter and a resulting formation of a fat crystal network in the bottom nougat layer that presumably blocks the fat migration pathway due to microstructural changes [27,28]. At this particular time, chocolate hardness was almost similar to the hardness of chocolate on top of the industrially tempered control nougat. Using chocolate hardness as an indicator, it was not possible to detect further changes after that period of time. Except for nougat #A seeded with 1% cocoa butter crystals, analysis of variance with subsequent post-hoc tests revealed that the hardness of the chocolate layer at the end of storage was significantly higher than its initial hardness. The more pronounced hardness increase of the nougat #D sample can be attributed to the lower amount of nut oil that was present in this system. As regards nougat with 2% seed crystals, chocolate on top of it was even harder than the control immediately after production of the layer models. This system remained stable during approx. 4 weeks but, subsequently, showed a significant trend toward time-dependent softening. Although the investigated systems are not fully comparable, the mechanism behind a reduced nut oil migration is presumably similar to that observed with respect to oil separation in spreadable nougat creme [29,30], namely that the formation of some sort of fat crystal network in the nougat significantly lower filling oil mobility.

**Figure 3:**
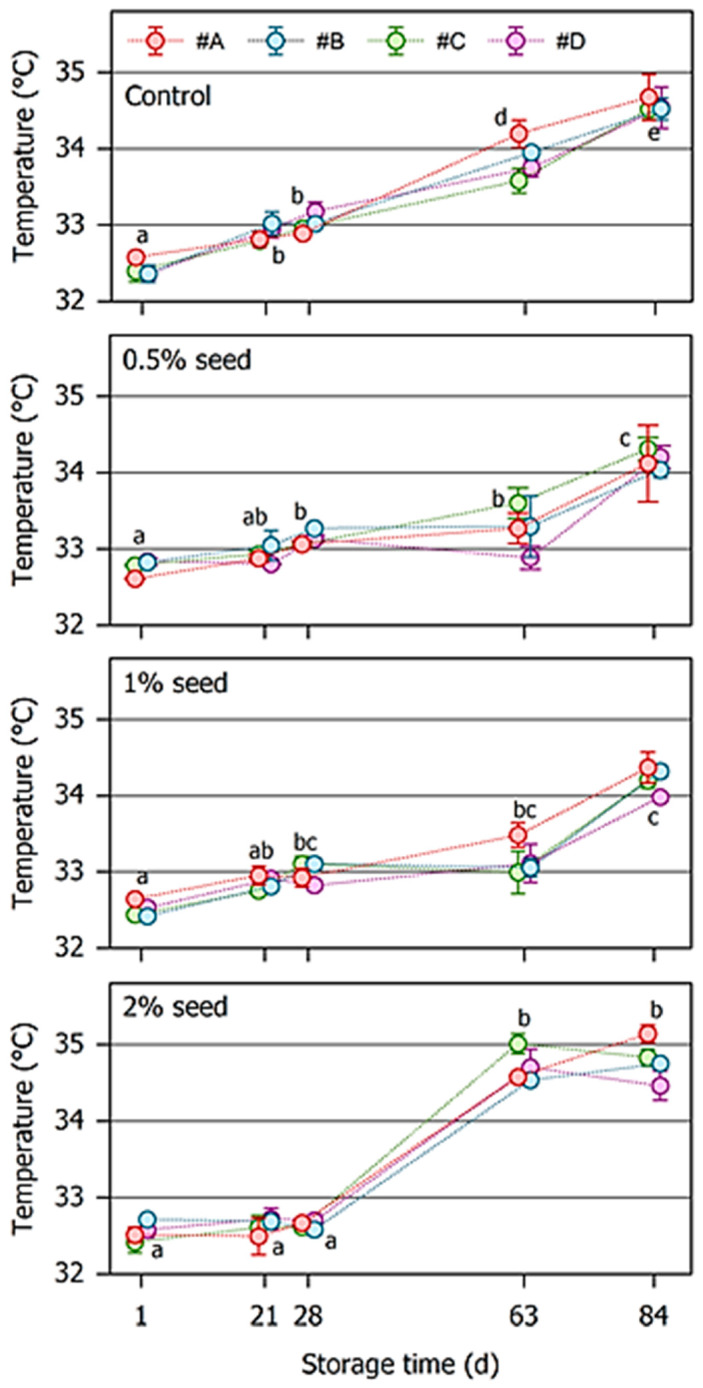
Development of the melting peak temperature of chocolate on top of the nougat/chocolate layer models during storage at 23 °C for 84 days. Four different nougats (#A, #B, #C, #D) were either tempered using the stir/shear technique (control) or seeded with different amounts of cocoa butter crystals. For the sake of clarity, symbols are slightly shifted along the x-axis. Individual data are arithmetic mean ± half deviation range of duplicate measurements. Mean values after different storage times labeled with different letters differ significantly (*p* < 0.05).

Figure 3 depicts the effects of nougat crystallization on storage-induced changes of the cocoa butter melting peak temperature in chocolate taken from the surface of the samples. Immediately after production, the peak temperature ranged from 32.2–32.9 °C, which is typical for cocoa butter with ßV crystals [31,32]. In all control samples, the cocoa butter melting temperature increased continuously with ongoing storage and finally reached approx. 34.5 °C. This shift indicates that a significant fraction of ßV crystals transformed into the ßVI modification, which shows a typical melting temperature of approx. 35–36 °C. Their presence can be regarded as an indicator and expression of fat bloom [3,9,33].

This increase in the temperature of the melting peak was significantly lower in chocolate placed on top of the nougat seeded with cocoa butter crystals than in the control sample. This effect was especially observed when 0.5% or 1% crystals were added, and the respective delay in the shift of the peak temperature was evident at least for a storage period of 63 d. When 2% seeding crystals were used, the melting temperature was stable for at least 28 d.

**Figure 4:**
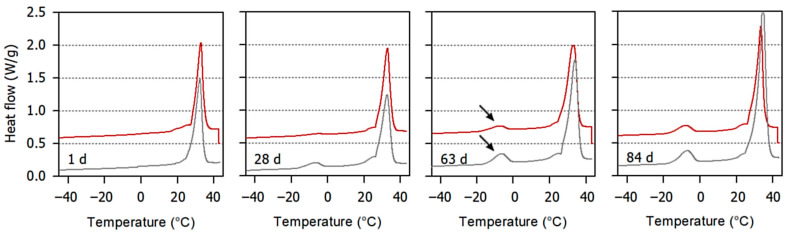
Average melting curves (endo up, *n* = 2) of chocolate on top of nougat stored for different periods of time (1, 28, 63, and 84 days). Grey lines, chocolate on top of control nougat; red lines, chocolate on top of nougat #A. The nut oil melting peak is marked by an arrow. For the sake of clarity, the red lines are shifted along the y-axis by 0.5 W/g.

Figure 4 depicts temperature-resolved thermograms of the chocolate placed on top of control nougat #A and those of chocolate on nougat #A that was seeded with 1% cocoa butter crystals. On the day after producing the layer models, the progress of the heat flow in the samples was almost identical. After storage for 21 d the presence of a compound with a melting temperature of approx. −7 °C, obviously resulting from a small amount of hazelnut oil being present, started to become visible especially in the chocolate of the layer models with the control nougat. When plotted against time, the phase transition enthalpy corresponds to the area of the heat flow peak.

For a storage time of 28 d and 84 d, the specific melting enthalpy of the chocolate surface as affected by the type of nougat and by the nougat crystallization method is outlined in Table 3. The relatively large deviation between some of the duplicate measurements can again be attributed to local effects at the surface where the samples were taken. However, it is, on average, evident from the data that, during the first period of storage, the seeding of nougat with cocoa butter crystals significantly reduced the migration of nut oil to the chocolate surface. This result is in good agreement with the melting peak data presented in Figure 3: a higher nut oil melting enthalpy in chocolate can be regarded to trigger the transformation of cocoa butter crystal morphology from ßV to ßVI with the accompanying shift in melting temperature. After long-term storage, the stabilizing effect of the seeding of nougat with cocoa butter crystals diminishes, and so do the significances of the differences.

In three sets of sensory discrimination tests, pure nougat seeded with 0.5% or 1% cocoa butter crystals was tested against the control prepared by the stir/shear technique and, in addition, against each other. In all cases, the number of correct identifications was seven at a maximum which means that the sensory panel could not discriminate the samples at an error probability level of *p* < 0.10.

**Figure 5:**
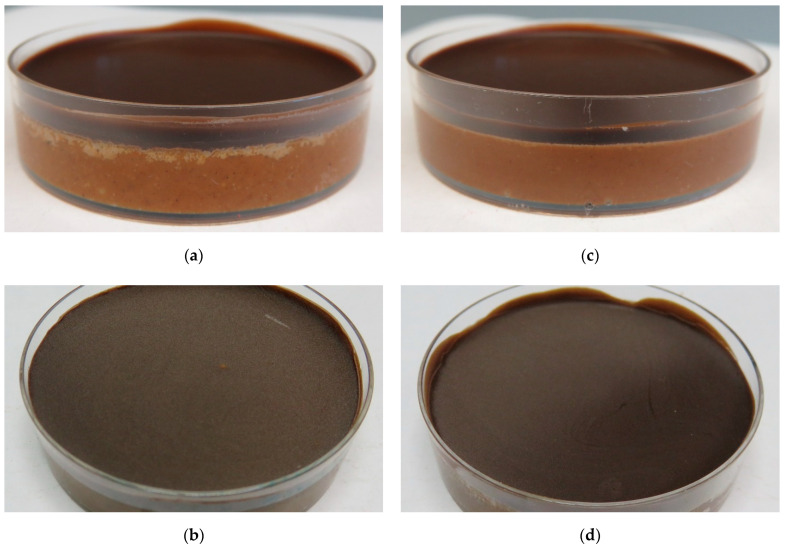
Layer models after 28 days storage at 23 °C showing enhanced fat bloom at the interface between nougat and chocolate (**a**) and at the chocolate surface (**b**), and corresponding systems with nougat #A seeded with 1% cocoa butter crystals: interface between nougat and chocolate (**c**) and chocolate surface (**d**).

Figure 5 finally shows the appearance of the nougat #A control layer models and, exemplary, the layer model with the same nougat seeded with 1% cocoa butter crystals. The photographs clearly show the differences in fat bloom intensity which is visible at the surface of the top layer chocolate and, especially, at the interface between the nougat layer and the chocolate.

## 4. Conclusions

The current study shows that, under controlled conditions, the pre-crystallization of nougat by seeding with cocoa butter crystals may be considered appropriate for improving the physical storage stability of pralines and delaying the onset of fat bloom. In the layer models used in the experiments, the thickness of the chocolate layer was uniform across the tested surface. Such a uniform chocolate shell thickness can be achieved when molding techniques such as the frozen cone technology are used. It needs, however, to be tested in further studies whether the stability of filled systems can also be improved when shell thickness variations, resulting from standard molding techniques such as form inverting or spinning, are present.

## Figures and Tables

**Table 1:** Formulation of the nougat samples (g/100 g) used in this study.

Ingredient	Nougat #A	Nougat #B	Nougat #C	Nougat #D
Hazelnuts	44.2	47.0	38.0	30.0
Sucrose	39.3	42.0	49.0	49.0
Cocoa mass	5.0		8.0	7.0
Milk solids	3.0			4.5
Cocoa butter	8.5	11.0	5.0	8.5
Sunflower lecithin			<0.1	<1.0
Vanilla extract				<0.1

**Table 2:** Fat distribution and physical properties of base nougat. Mean values with different superscripts in a column indicate statistical differences (*p* < 0.05).

Nougat	Total Fat (g/100 g) ^1^	Mobile Fat (%) ^2^	Yield Stress (Pa) ^2^	Viscosity (Pa.s) ^2^	Hardness (N) ^3^
#A	37.6	27.2 ± 0.3 ^a^	10.78 ± 0.07 ^b^	1.75 ± 0.02 ^b^	0.95 ± 0.12 ^b^
#B	44.0	25.5 ± 0.8 ^a^	3.78 ± 0.05 ^c^	1.43 ± 0.04 ^c^	1.31 ± 0.16 ^b^
#C	37.0	23.1 ± 1.1 ^ab^	10.25 ± 0.17 ^b^	3.25 ± 0.04 ^a^	1.24 ± 0.24 ^b^
#D	36.0	18.5 ± 1.0 ^b^	12.90 ± 0.09 ^a^	3.87 ± 0.03 ^a^	3.76 ± 0.32 ^a^

^1^ Data from manufacturer specifications; ^2^ Data is arithmetic mean ± half deviation range (*n* = 2); ^3^ Data is arithmetic mean ± standard deviation (*n* = 8).

**Table 3:** Effects of crystal seeding on specific enthalpies (J/g) of the nut oil melting peak in the chocolate on top of nougat layers after 28 or 84 days of storage at 23 °C. Mean values with different superscripts in a column indicate statistical differences (*p* < 0.05).

Nougat Seeding	Nougat #A ^1^	Nougat #B	Nougat #C	Nougat #D
Storage time: 28 days				
Control	1.83 ± 0.25 ^a^	2.02 ± 0.30 ^a^	1.64 ± 0.27 ^a^	0.73 ± 0.09 ^a,b^
0.5% seed crystals	1.07 ± 0.18 ^b^	1.56 ± 0.14 ^a^	0.71 ± 0.05 ^b^	1.09 ± 0.32 ^a^
1% seed crystals	1.09 ± 0.02 ^b^	1.58 ± 0.28 ^a^	0.76 ± 0.12 ^b^	0.28 ± 0.09 ^b^
2% seed crystals	0.96 ± 0.12 ^b^	0.42 ± 0.04 ^b^	0.37 ± 0.04 ^b^	0.32 ± 0.12 ^b^
Storage time: 84 days				
Control	9.21 ± 0.73 ^a^	8.77 ± 0.23 ^a^	7.34 ± 0.77 ^a^	4.65 ± 0.18 ^a^
0.5% seed crystals	6.58 ± 0.44 ^b^	4.45 ± 0.36 ^b^	7.37 ± 0.04 ^a^	4.73 ± 0.52 ^a^
1% seed crystals	6.05 ± 0.46 ^b^	8.68 ± 0.62 ^a^	7.38 ± 0.58 ^a^	3.66 ± 0.54 ^a^
2% seed crystals	9.03 ± 0.44 ^a^	7.78 ± 0.89 ^a^	6.90 ± 0.07 ^a^	4.51 ± 0.24 ^a^

^1^ Data is arithmetic mean ± half deviation range (*n* = 2). Mean values in a data block marked with different superscripts (a,b) differ significantly (*p* < 0.05).

## Data Availability

The data presented in this study are available on request from the corresponding author.

## Supplementary Materials

The following are available online at https://www.mdpi.com/article/10.3390/foods10051056/s1, Figure S1: Melting curves (duplicate measurement) of the ßV cocoa butter seed powder used in this study, Figure S2: Light microscopy images of the ßV cocoa butter seed powder used in this study.
