# Supplementary material for: Pre-Crystallization of Nougat by Seeding with Cocoa Butter Crystals Enhances the Bloom Stability of Nougat Pralines
Source: Foods. 2021 May 11;10(5):1056. doi: 10.3390/foods10051056 (PMC8151285; doi:10.3390/foods10051056)
Supplement: Supplementary file 1 [file foods-10-01056-s001.zip › foods-1183697-supplementary.pdf]

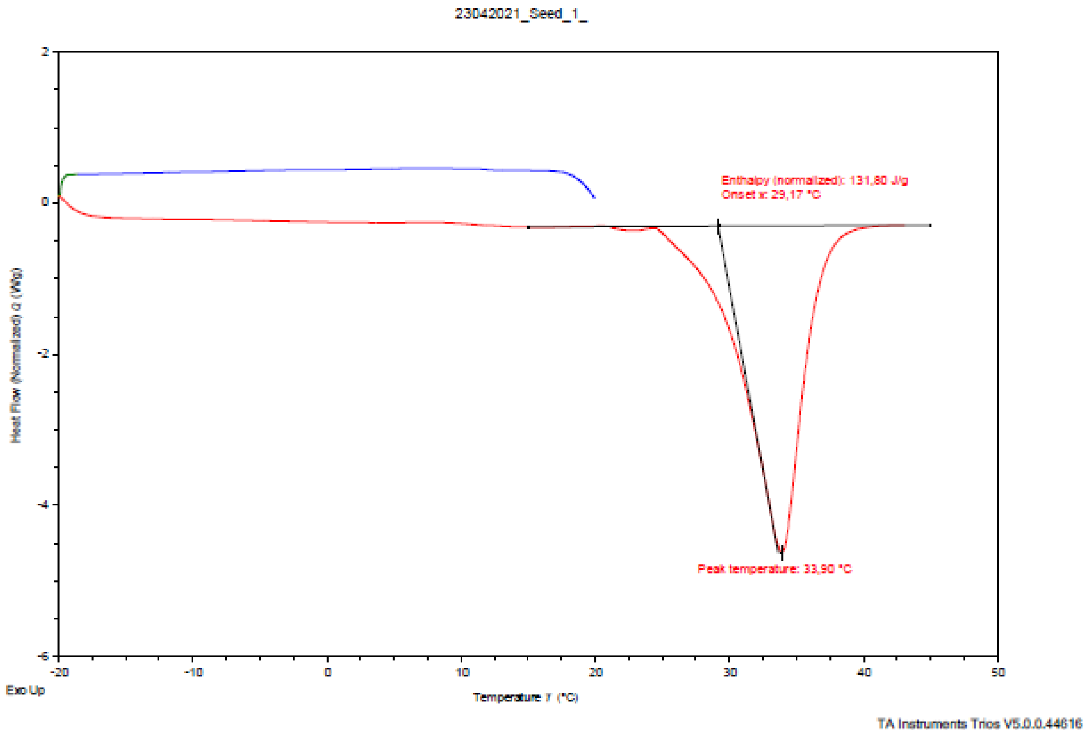

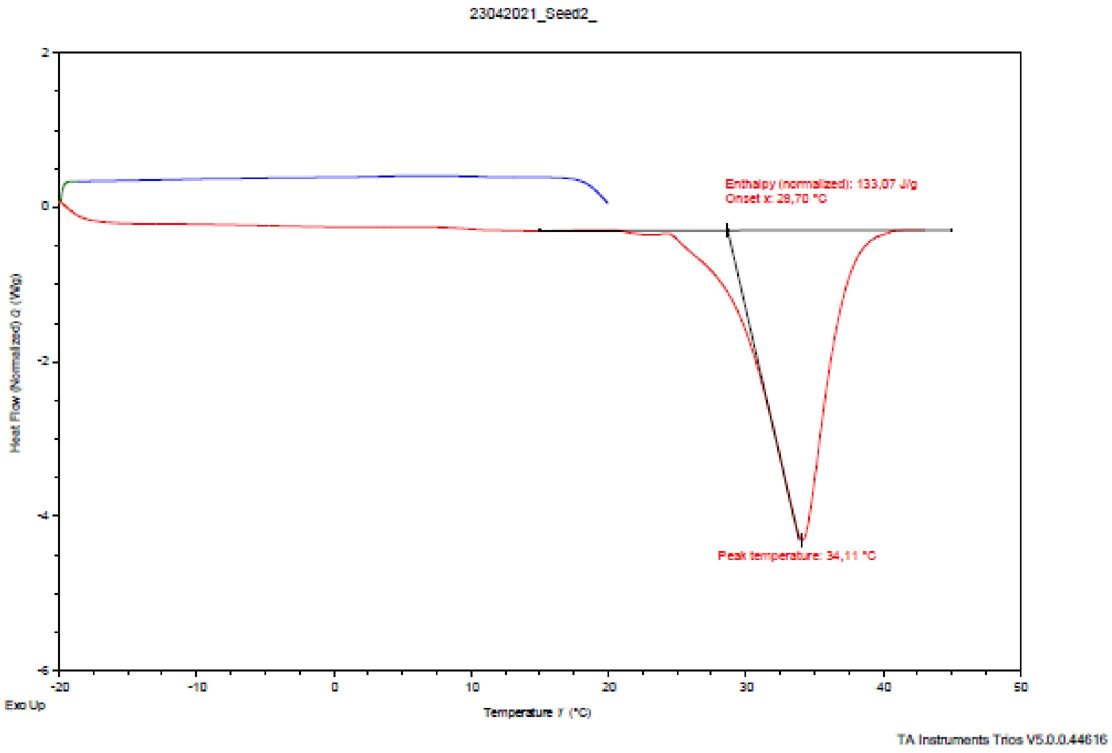

Figure S1: Melting curves (duplicate measurement) of the ßV cocoa butter seed powder used in this study.

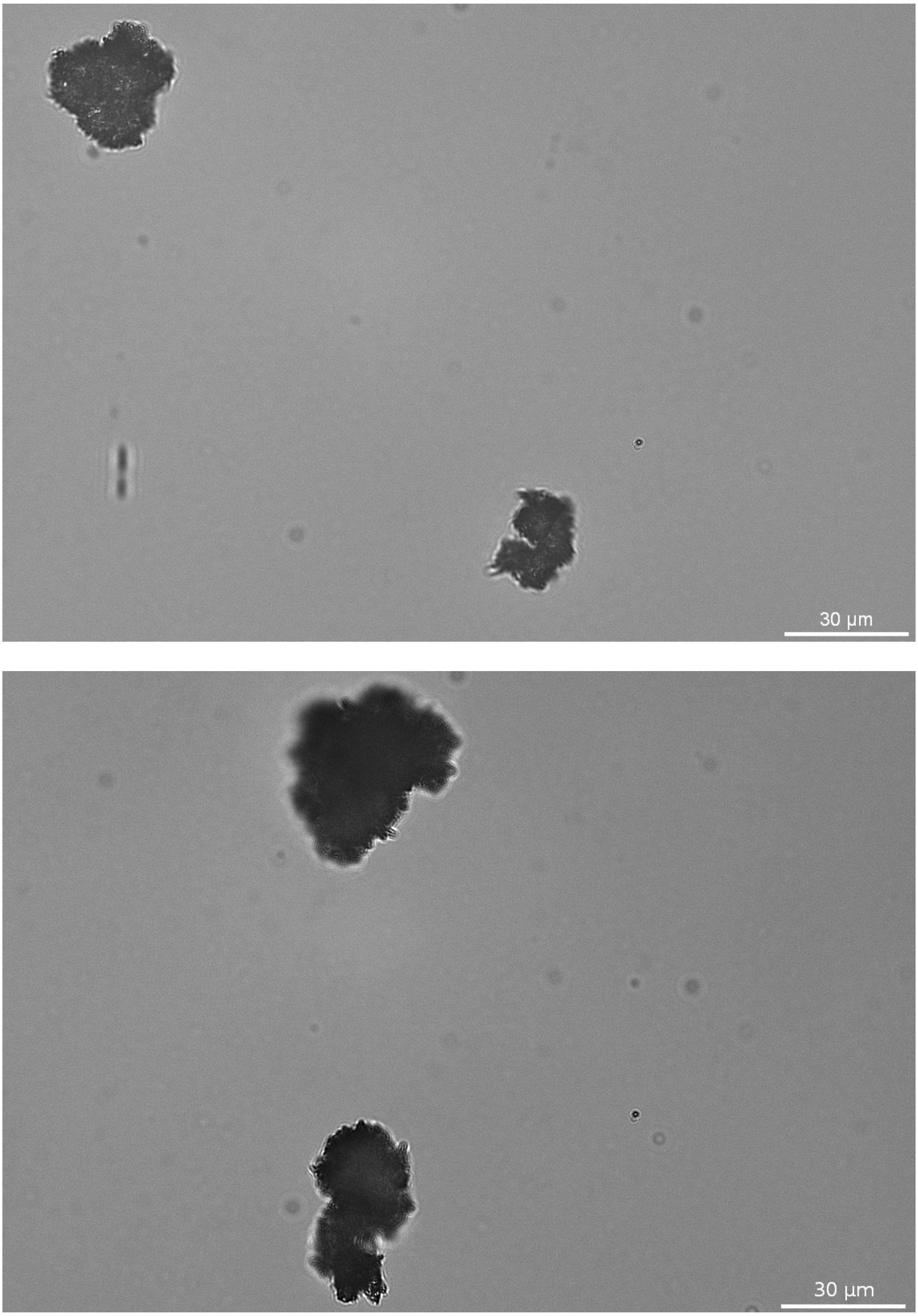

Figure S2: Light microscopy images of the ßV cocoa butter seed powder used in this study.
